# Fine tuning of vitamin D receptor (VDR) activity by post-transcriptional and post-translational modifications

**DOI:** 10.18632/oncotarget.15697

**Published:** 2017-02-25

**Authors:** Ondrej Zenata, Radim Vrzal

**Affiliations:** ^1^ Department of Cell Biology and Genetics, Faculty of Science, Palacky University, Olomouc, Czech Republic

**Keywords:** miRNA, phosphorylation, ubiquitination, sumoylation, VDRB1

## Abstract

Vitamin D receptor (VDR) is a member of the nuclear receptor (NR) superfamily of ligand-activated transcription factors. Activated VDR is responsible for maintaining calcium and phosphate homeostasis, and is required for proper cellular growth, cell differentiation and apoptosis. The expression of both phases I and II drug-metabolizing enzymes is also regulated by VDR, therefore it is clinically important.

Post-translational modifications of NRs have been known as an important mechanism modulating the activity of NRs and their ability to drive the expression of target genes. The aim of this mini review is to summarize the current knowledge about post-transcriptional and post-translational modifications of VDR.

## INTRODUCTION

The vitamin D receptor (VDR; NR1I1) was discovered in 1969 as a protein activated by the hormonal form of cholecalciferol 1α,25-dihydroxyvitamin D_3_ (1,25(OH)_2_D_3_ or calcitriol) [1]. Today it is known that the VDR is a member of the superfamily of nuclear steroid/thyroid hormone receptors present in the cytoplasm and nucleus. Earlier observations describing VDR as a receptor with predominant nuclear localization were disproved by using a fluorescently labeled ligand [2] and this was later confirmed by confocal microscopy [3]. Following ligand binding, the VDR forms a heterodimer with retinoid X receptor (RXR; NR2B1) and regulates the expression of genes *via* binding to vitamin D responsive element (VDRE; short nucleotide sequence with direct, everted or inverted repeats) found in their regulatory regions. Vitamin D receptor gene is located on chromosome 12. The precise physical localization in the centromeric region 12cen-q12 was confirmed by fluorescent *in situ* hybridization in 1999 [4].

**Figure 1:**
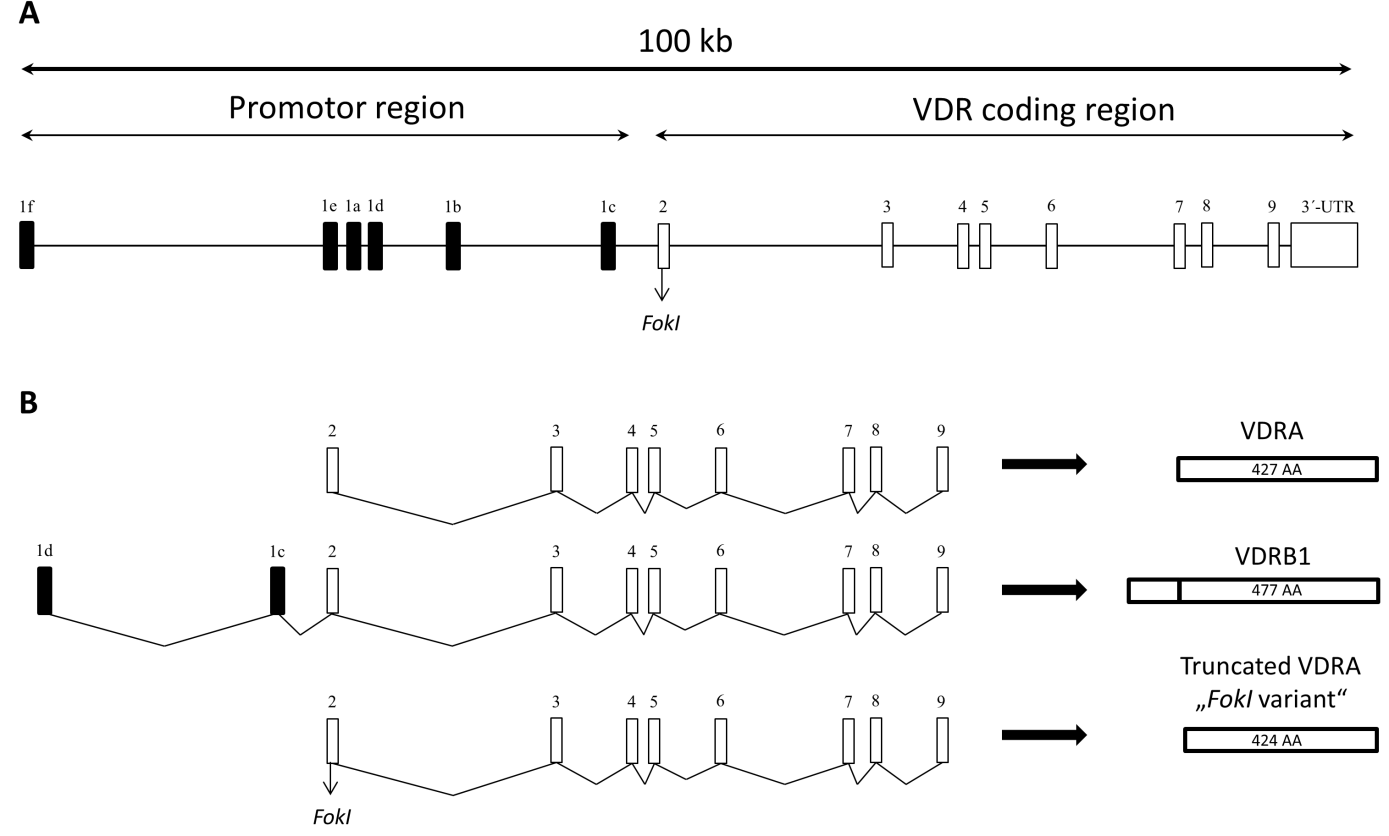
A. Schematic representation of human VDR gene locus, which is spread on the area about 100 kb VDR gene consists of 14 exons, six of them being variants of exon 1, which participates on alternative splicing. Exons 2-9 are common to all known VDR protein isoforms. **B**. Schematic demonstration of VDR protein composition. Most common variant of VDR is called VDRA. It consists of exons 2-9 and has 427 amino acids (AA). VDRB1 variant is elongated on N-terminal domain by two exons (1d and 1c) and has 477 AA. Truncated VDRA is caused by *FokI* polymorphism, which is present at translation initiation codon, and has 424 AA.

The gene consists of 14 exons of approximately 100 kb and can be divided into two main regions (Figure 1A). The first exon is located in the promotor region and has six variants (a - f) important for alternative splicing of VDR. Exons 2 - 9 present in the coding region are common for all 14 known transcripts [5–7]. To this date, only three isoforms (two are made by alternative splicing, one by polymorphism in translation initiation codon) of VDR were discovered in human cells or cell lines (Figure 1B). The most emerging form is VDRA consisting of 427 amino acids (48 kDa). Second isoform, called VDRB1, is elongated on N-terminal domain about 50 amino acids (477 amino acids; 54 kDa) by start site (ATG) in exon 1d (VDRA has start site in exon 2) and was found in human kidney, intestinal and kidney epithelial cell lines [5, 8]. This elongation enables different reaction to ligands (calcitriol or lithocholic acid) in diverse types of tissue, suggesting that VDRA and VDRB1 activation is ligand- and tissue-specific [9, 10]. Third isoform forms due to the *FokI* polymorphism of VDR. *FokI* (defined by the restriction enzyme) is present at translation initiation codon and results in a formation of shorter VDR that has higher transcriptional activity than full length VDR [11].

Vitamin D receptor is activated by calcitriol, formation of which is initiated in the skin. UV-B radiation converts 7-dehydrocholesterol into previtamin D_3_, which is quickly transformed into vitamin D_3_ (cholecalciferol, it can be taken from diet). Another dietary available form, vitamin D_2_ (ergocalciferol), then follows the same metabolic fate as vitamin D_3_. Vitamin D (D represents D_2_ or D_3_) is hydroxylated on position 25 by microsomal P450 enzymes (CYP27A1, CYP2R1, CYP2J2, CYP2J3 and CYP3A4) to 25-hydroxyvitamin D [25(OH)D] mostly in human liver. Due to the stability in blood and a long circulating half-life lasting 2 weeks, 25(OH)D is widely accepted as an indicator of vitamin D status [12]. Normal physiological level (vitamin D sufficiency) is defined as 25(OH)D > 30 ng/ml (75 nmol/L) and vitamin D deficiency as 25(OH)D < 20 ng/ml (50 nmol/L) [12]. 25(OH)D is biologically inactive and must be converted in the kidneys by 25(OH)D-1α-hydroxylase (CYP27B1) into 1,25-dihydroxyvitamin D [1,25(OH)_2_D], active form of vitamin D. The overall schema of synthesis is clearly and representatively presented in other papers [13, 14]. Regarding the dietary sources of vitamin D (D_2_ or D_3_), older study demonstrated 1.7-times greater efficacy for vitamin D_3_ then vitamin D_2_ in maintaining the 25(OH)D status [15]. However, recent research brought the proof that vitamin D_2_ is equally effective [16]. Moreover, binding affinities of 1,25(OH)_2_D_2_ (ergocalcitriol) or 1,25(OH)_2_D_3_ (calcitriol) to VDR or transactivation potencies on target genes are almost comparable [17, 18].

VDR is involved in bone and calcium homeostasis, cell differentiation, immunomodulation and control of other hormonal systems [19]. The role of VDR in immunity was reported in many clinical studies. High doses of vitamin D helped to prevent illness caused by influenza [20, 21] or other respiration infections [22]. There are also evidences suggesting that vitamin D supplementation can increase the effects of anti-tuberculosis cure. Indeed, 1,25(OH)_2_D enhances innate immunity by increasing the expression of antimicrobial peptides including cathelicidin, a peptide involved in elimination of *Mycobacterium tuberculosis* [23, 24]. Moreover, VDR positively stimulates the expression of gene for phospholipase C-γ1 (PLC- γ1) in naive human T-cells. This gene plays a key role in classical T-cell antigen receptor signaling and T-cell activation [25]. The importance of vitamin D was demonstrated even on muscle strength predominantly in the elderly people [26] or femoral whole-bone strength in older men [27]. Furthermore, it has been shown that 1,25(OH)_2_D has anticancer effect against prostate [28, 29] and breast cancers [28, 30].

As in many other genes, there were described polymorphisms in the promotor and coding gene regions of VDR as well. For instance, Fang et al. found haplotype alleles in promotor region and in the 3´-untranslated region (3´-UTR), which are strongly associated with an increased risk of bone fracture being independent on age, sex, height, weight or bone mineral density of subjects [31]. Recently, it was reported the relationship between *FokI* polymorphism of VDR and susceptibility to chronic periodontitis [32], AIDS disease [33] or more active immune system for the shorter *FokI*-VDR variant [34].

A separate chapter is a role of VDR in cancer. On the one hand, it was demonstrated that vitamin D supplementation possess anti-cancer activity in mouse xenografts of breast and prostate cancer [28] and that epidermal growth factor receptor mutant lung cancer is a vitamin D-responsive disease [35]. In addition, epidemiological studies usually find inverse relationship of vitamin D status and risk of breast cancer. However, in general, the results are often conflicting, which is expected to be due to the differences in methods for selecting cases and controls, dietary intake data collection tools and referent time period [36]. On the other hand, recent meta-analysis found an association between *FokI* polymorphism and the susceptibility to prostate cancer in Caucasian population [37]. Thus, VDR status can be a double-edge sword in relation to cancer.

However, the different effects of VDR action in diverse types of tissues cannot be explained just by polymorphisms of VDR, splicing variants or tissue specific environment. Thus, post-transcriptional and post-translational modifications (PTMs) of VDR can represent another level of VDR activity regulation.

## POST-TRANSCRIPTIONAL REGULATION OF VDR BY MIRNA

MicroRNAs (miRNAs) are a class of short (about 22 nucleotides) non-coding RNAs with wide gene regulatory activity, which causes complementary mRNA degradation or translation repression *via* binding to the 3´-UTR of mRNA [38]. They are necessary for ensuring fundamental processes in the human body and their deregulation has been associated with several diseases, including cancer [39].

The first investigated miRNA regulating VDR is the mature miR-125b produced from two precursors (miR-125-b1 and miR-125-b2) localized in chromosome regions 11q24.1 and 21q11.2 [40]. It was demonstrated, that region 11q23-24 is frequently deleted in breast, lung and ovarian cancers [41, 42] and the chromosomal region 21q11-21 in breast, esophagus, ovary, lung and stomach cancers [43].

**Figure 2:**
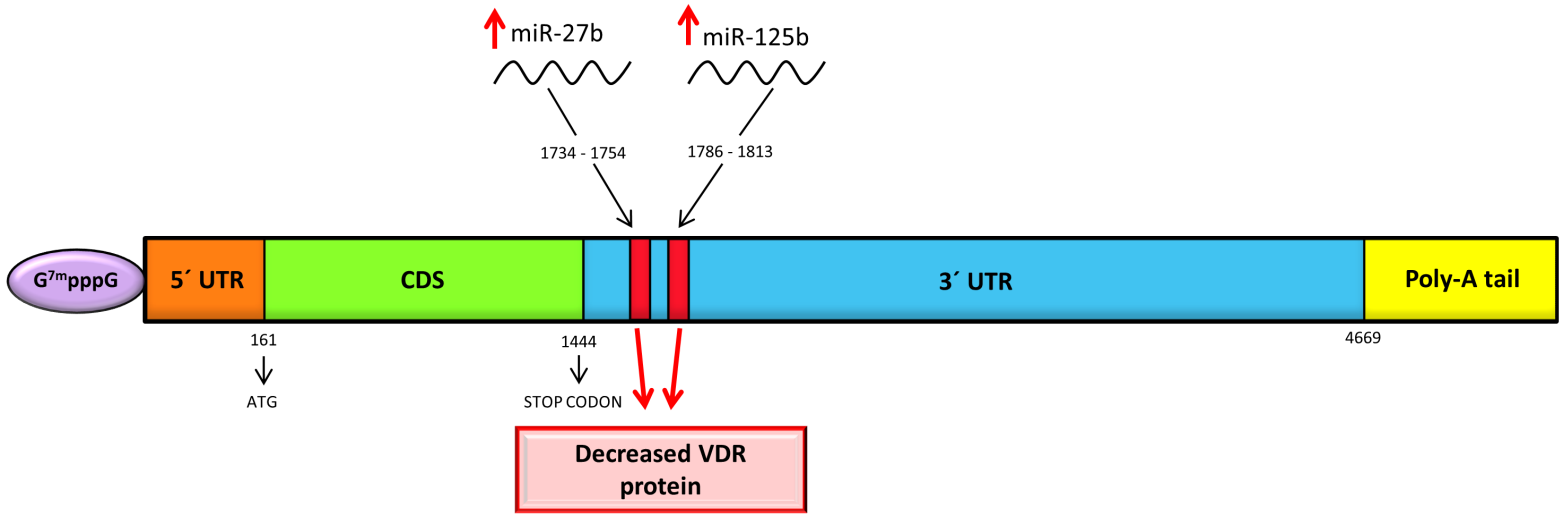
Schematic representation of human VDR mRNA with miRNAs binding sites at 3´-UTR Both miRNAs bind to 3´-UTR, which results in the decreased VDR protein.

Mature miR-125b binds to miR-125b recognition element (MRE 125b; highly conserved sequence found among species) in target mRNAs and regulates their amounts or attenuates translation without implication on mRNA. MRE125b is located in the 3´-UTR in human VDR site from +1786 to +1813 (Figure 2) with the core sequence of CUCAGGG (essential sequence for the binding of the miR-125b to the mRNA) [40]. Mohri et al. demonstrated a negative influence of miR-125b overexpression on VDR protein level (40 % reduction of protein in contrast to untreated cells), with no effect on VDR mRNA level. *Vice versa*, after reduction of miR-125b, VDR protein level was increased by 130%, but still without any effect on mRNA level [40]. This increase of VDR may result in the augmentation of the antitumor effect of calcitriol in cancers mentioned above.

However, it is an interesting fact that this miRNA targets the mRNA of CYP24, a VDR-regulated gene responsible for metabolic degradation of 1,25(OH)_2_D. In the study by Komagata et al., it was found that increased expression of miR-125b resulted in the decreased CYP24 protein and CYP24-mediated enzymatic activity [44]. Thus, possible effect of CYP24 inhibition and calcitriol treatment in case of breast cancer patients may bring synergistic benefits.

Another miRNA with a direct effect on the VDR is miR-27b. Increased level of miR-27b (with transfected miR-27b mimic) resulted in the lower level of VDR protein (miR-27b targets 3´-UTR of VDR) (Figure 2), with no effect on mRNA of VDR in human lung fibroblasts (MRC-5) cells [45]. Reduced level of miR-27b (with miR-27b inhibitor) caused opposing effects, but still without any change in VDR mRNA level. Moreover, by employing luciferase reporter assay it was demonstrated that miR-27b directly targets 3´-UTR of VDR [45].

Furthermore, the influence of VDR on protein Dicer essential for the maturation of pre-miRNA to miRNA is also worth mentioning. The VDRE was found in the promoter region of Dicer gene. Upon binding of VDR/RXR to VDRE, the amount of Dicer was increased on both levels, mRNA and protein. However, upregulation of Dicer was observed only at high concentration of 1,25(OH)_2_D (1 mM) [46]. Among the most affected miRNAs were miR-22 (miR-22 regulates telomerase necessary for many types of cancer), miR-296-3p and miR-498. All three miRNAs are involved in anticancer actions and were upregulated [46]. Nevertheless, this finding has probably no serious implication in clinical practice since serum 25(OH)D levels above 150 ng/mL are considered as vitamin D intoxication [47]. Moreover, due to the tight control of 1,25(OH)_2_D formation, serum levels similar to that used in the study mentioned [46] are not reachable.

## POST-TRANSLATIONAL MODIFICATIONS (PTMS) OF VDR

### Phosphorylation

Phosphorylation is the addition of phosphoryl group (PO_3_^2−^) to a molecule (in our case to VDR). It plays a significant role in proteins regulation by altering their function and activity. The first enzymatic phosphorylation of proteins was described in 1953 by Burnett and Kennedy, who used radioactively labeled ATP (adenosine triphosphate) to demonstrate a new type of enzymatic action [48]. Several members of the steroid/thyroid hormone receptor superfamily are known to be phosphorylated, including glucocorticoid receptor [49], estrogen receptor [50] or thyroid hormone receptor [51], and VDR is not an exception. The first evidence of phosphorylated VDR came in 1985. It was demonstrated that 1,25(OH)_2_D stimulated the phosphorylation of VDR in mouse fibroblast 3T6 cells [52] and the same mechanism was observed for chicken VDR [53].

**Figure 3:**
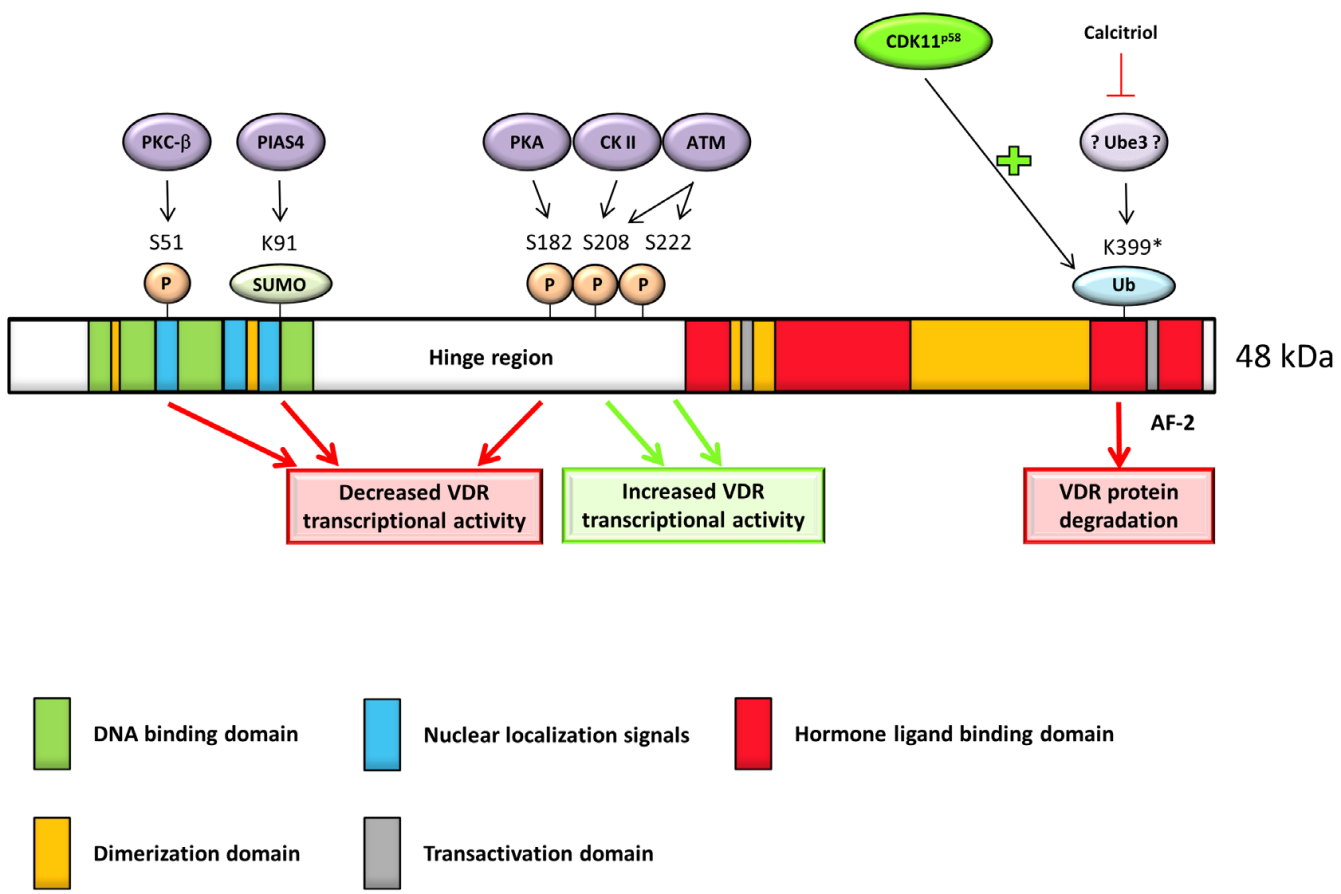
Full length VDR protein has five main parts essential for its function: DNA binding domain allows DNA binding; nuclear localization signals direct the receptor into the nucleus; hormone/ligand-binding domain allows ligand binding; dimerization domain is necessary for heterodimerization with RXR and transactivation domain interacts with coactivators Phosphorylation of S208 has direct influence on VDR heterodimerization, while phosphorylation of S51 attenuates transcription activity. Sumoylation of K91 and phosphorylation of S182 have negative effect on VDR transcription activity. Phosphorylation of S222 has positive effect on VDR activity. There are indirect proofs that potential ubiquitination site on position K399 target VDR to degradation (* = indirect proof) and this effect is protected by calcitriol *via* suppression of so far unknown ubiquitin E3 ligase. CDK11^p58^ promotes VDR ubiquitination with subsequent repression of VDR activity and stability.

Several years later, it was discovered that serine at position 51 (in sequence RRS_51_MKRK located between the two zinc fingers) in VDR is a substrate for protein kinase C-β (PKC-β) (Figure 3) [54]. A point mutation (serine to glycine; AGC to GGC) in this region led to abolished PKC-β-catalyzed phosphorylation compared with the phosphorylation in wild-type VDR (*in vitro* and *in vivo*). Moreover, this amino acid change resulted in the decreased transcriptional activity of the VDR after 1,25(OH)_2_D treatment (10 nM for 48 h). This data suggested positive role of phosphorylation at serine 51 in 1,25(OH)_2_D-dependent transcriptional activation of VDR since de-phosphorylation, which can be equalized to phospho-deficient mutation, resulted in the decrease of VDR activity. Other tested serine residues (Ser119 and Ser125 changed to glycine and alanine, respectively) were not confirmed as phosphorylation sites of PKC-β [54].

Furthermore, the type of amino acid seems to be very important as it was revealed by Hsieh et al. two years later [55]. Replacement of serine with glycine (S51G), aspartic acid (S51D, mimic negative charge) or threonine (S51T) led to the decrease of transcription activity (by 65%, 90% and 55%, respectively) compared to the wild type VDR in 1,25(OH)_2_D-treated cells [55], i.e. no matter of phospho-deficient of -mimic mutation introduction. Moreover, VDR mutant with alanine (S51A) eliminated PKC-β phosphorylation but completely retained wild-type VDR transactivation capacity and its binding ability to the VDRE (only S51A and S51T mutants were confirmed to form complex with the VDRE on the observable level). Moreover, incubation of *E. coli*-expressed VDR with PKC-β elicited significant decreased ability to bind into the VDRE [55]. This observation brings the proof that phosphorylation at position serine 51 has negative influence on VDR transcription activity. However, it is important to keep in mind that purified VDR from *E. coli* has no other post-translation modifications (sumoylation, ubiquitination or other phosphorylations) which can play together an important role in VDR transcription activity or ability to bind into the VDRE. This also suggests that the secondary structure of region around serine at position 51 is more important for function of VDR than the phosphorylation itself. Probably, delicate balance among amino acids charges in this area located between two zinc-fingers of DNA-binding domain is essential.

Interestingly, PKC-β was found to be positively regulated by VDR. Treatment of HL-60 cells (human promyelocytic leukemia cell line) with 1,25(OH)_2_D resulted in a 4.2-fold increase of mRNA PKC-β and 3.8-fold increase in its transcription rate [54, 56]. This suggested positive autoregulatory loop for increased activity of VDR by 1,25(OH)_2_D.

Another serine phosphorylation is described at position 208. Unlike serine at position 51 (Figure 3), this phosphorylation is caused by casein kinase II (CK-II) localized in sequence NLDLS_208_EEDSDD, typical CK-II consensus recognition site, and it is enhanced by 1,25(OH)_2_D [57, 58]. Replacement of serine with glycine led to a slightly decreased 1,25(OH)_2_D-stimulated transcriptional activity [59]. It corresponds to the fact that phosphorylation at this site is not obligatory for VDR action as at position 51 [54, 59]. Mechanism of positive effect of phosphorylation at position 208 was discovered later. Barletta et al. found that response of VDR to 1,25(OH)_2_D was enhanced by okadaic acid (phosphatase inhibitor), not due to up-regulation of VDR or enhanced VDR-RXR interaction with the VDRE, but by stimulation (3- to 4- fold) of protein-protein interaction between VDR and DRIP205 (a subunit of the vitamin D receptor-interacting protein) [60]. These results were later confirmed by another research group which clearly demonstrated positive effect of phosphorylation at position 208 for DRIP205 [61]. This data indicated a specific role for both phosphorylation sites.

The first evidence dealing with the involvement of protein kinase A (PKA) in ligand-dependent VDR-mediated transactivation was reported in 1993 [62]. Phosphorylation of human VDR co-transfected into COS-7 cells with PKA led to PKA-dependent attenuation of 1,25(OH)_2_D-induced transcriptional activity after 24h [62]. The similar results were obtained for HeLa and Saos-2 cell line. Significant decrease of 1,25(OH)_2_D-stimulated VDR transcription activity was found in the presence of 8-bromoadenosine cyclic 3´,5´-monophosphate (8 bromo-cAMP; activator of PKA, resistant to degradation by cyclic AMP phosphodiesterase) in above mentioned cell lines with co-transfected VDR and reporter gene construct. In the HeLa and Saos-2 cell lines, the effect of 1,25(OH)_2_D was suppressed to 61% and 78% after 36 h, respectively [63]. However, point mutations in possible PKA phosphorylation sites (serine 172 and threonine 175, both replaced by glycine) resulted in no change of VDR response to 1,25(OH)_2_D [63]. Four years later, serine at position 182 was discovered as a target of PKA (Figure 3) in the sequence SGDS_182_SSSCSDHC. Phosphorylation at this position has negative impact on heterodimerization with RXR and results in the decreased transactivation by 1,25(OH)_2_D [64]. Despite of evidence about negative influence of PKA mediated phosphorylation, Huening et al. provided the proof about positive effect of PKA on rat VDR [65]. Parathyroid hormone or cAMP (both activators of PKA) up-regulated VDR and augmented the 1,25(OH)_2_D-dependent induction of CYP24A1 and osteopontin after 9 h treatment in UMR-106 cell line (rat osteosarcoma). Induction of inducible cAMP early repressor, serving as dominant negative regulator of cAMP-induced transcription, repressed enhancement by PKA activators [65]. This data show different regulation between human and rat VDR. This may not be so much surprising since BLAST analysis between human (NP 001017535.1) and rat (NP 058754.1) VDR shows only 90.31% identity in amino acid sequence.

Another kinase involved in the regulation of PTMs of VDR and in clear relationship to cancer is ATM (ataxia telangiectasia mutated). This DNA-damage response kinase is recruited to DNA double strand breaks where it phosphorylates H2A histone family member X (H2Ax). However, in 2011 it was demonstrated that mutation of putative phosphorylation sites of VDR (serine 208 and 222) impaired the ability of ATM to enhance VDR transactivation activity [66]. Moreover, since ATM kinase was induced by 1,25(OH)_2_D, the positive feedback loop between carcinogens or oncogene stress and cancer prevention by vitamin D was suggested.

So far, this is the complete list of known kinases affecting VDR activity with identified or putative phosphorylation sites, by the end of 2016. However, there exist indications that other kinases could play a part. For instance, researchers found that activated c-Jun N-terminal kinase 1 (JNK1) physically and functionally interacts with VDR and positively regulates VDR expression at transcriptional and translational level resulting in the increased 1,25(OH)_2_D-mediated inhibition of colon cancer cell proliferation (HT29 cell line) [67]. Besides colon cancer, it was demonstrated that JNK1 cooperates also with p38 (another mitogen-activated protein kinase), activating VDR and increasing 1,25(OH)_2_D-dependent growth inhibition in breast cancer cells (MCF-7) [68]. This finding could be clinically important, since a widespread used sweetener sorbitol, an activator of JNK, is taken by many people from diet every day [69]. On the other hand, this might cancel the vitamin D-prevented amyloid-beta cytotoxicity [70], a pathological marker of Alzheimer's disease, due to the induced amyloid precursor protein (APP) phosphorylation by activated JNK in neurons [71]. Thus, tissue-specific phosphorylation of VDR can have beneficial as well as adverse impacts.

### Ubiquitination

The modification of the VDR by the covalent attachment of one or more ubiquitin molecules is termed ubiquitination. These small proteins (8.5 kDa) act usually as a recognition signal for 26S proteasome. The ubiquitin-proteasome pathway is the major route for the selective degradation of short-lived regulatory proteins in eukaryotic cells [72].

The first evidence about proteasome-mediated degradation of VDR was brought in 1998 [73]. It was shown that activation function 2 (AF-2; ligand-dependent domain localized on C-terminus of VDR (Figure 3) [73, 74]) domain of human VDR interacts with suppressor for gal 1 (SUG1, component of the 26S proteasome) in ROS17/2.8 cell line (rat osteosarcoma). This interaction targets VDR for proteasome-mediated degradation. Treatment with 26S proteasome inhibitors (MG132, beta-lactone) increased steady-state level of the VDR protein in the absence of ligand. On the other hand, in the presence of cycloheximide, ligand-bound VDR was degraded twice slower (half-life approximately 8 h) compared to ligand-free VDR (half-life approximately 4 h) [73]. While other receptors, such as aryl hydrocarbon receptor (AhR), estrogen receptor (ER) or mineralocorticoid receptor (MR), are targeted for degradation by ubiquitin-proteasome machinery in the presence of ligand [75–77], 1,25(OH)_2_D positively regulates amount of VDR protein (without altering to mRNA) in human keratinocytes by slowing down VDR degradation [78]. The same effect of protection by 1,25(OH)_2_D was observed in osteoblasts (hFOB, ROS 17/2.8 and primary mOSB cell lines) [79] and human CD4^+^ T Cells [80] but not in intestine cell lines (Caco-2, HT29, LS174 and mDuo) [79].

It seems that the main principle of this protection is heterodimerization with RXR and translocation to the nucleus after ligand binding. 1,25(OH)_2_D-bound VDR without the possibility of translocation (mutations in nuclear localization signal; R49W/R50G and K53Q/R54G/K55E) or heterodimerization (mutations in the dimerization interface; M383G/Q385A), is thus a target for polyubiquitination and 26S proteasome-mediated degradation [79]. The sequence required for polyubiquitination of VDR is possibly localized between amino acid residues 403 and 410 (CLSFQPEC), since deletion of this region diminished polyubiquitination of VDR in transfected Cos-1 cells [79]. However, there is no lysine residue required for covalent ligation of ubiquitin by E3 ligase within this sequence. Consequently, researchers assumed lysine on position 399 (Figure 3) as a possible target of an E3 ligase.

Recently, it has been shown that cyclin-dependent kinase 11, a 58 kDa protein (CDK11^p58^), is an interacting partner of VDR. Upon binding to VDR, CDK11^p58^ promotes VDR ubiquitination (Figure 3) leading to decreased level of protein and repressed VDR-dependent transcriptional activation. This data suggested CDK11^p58^ as a negative regulator of VDR activity and stability. However, the exact mechanism of promoting ubiquitination is still unclear [81].

### Sumoylation

Small Ubiquitin-like modifier (SUMO) is a family of small proteins covalently attached to lysine residues of other proteins in cells to modify their activity and function in process called sumoylation. This mechanism, essential for genome stability, was identified in mammals, where SUMO was found to be covalently linked to the Ran-GTPase activating protein (RanGAP1) [82, 83]. Several members of the nuclear receptor superfamily were also identified as targets of sumoylation, for example - estrogen receptor (ER) [84], androgen receptor (AR) [85], pregnane X receptor (PXR) [86] and peroxisome proliferator-activated receptor gamma (PPAR-γ) [87], with significant impact upon their function as transcriptional activators.

The first identification of VDR as a target of SUMO2 (in humans were confirmed 4 isoforms of SUMO differing in amino acid sequence [88]) was demonstrated in 2012 [89]. Sumoylation of VDR is facilitated by protein inhibitor of activated STAT 4 (PIAS4) having E3-ligase activity and serving as a potent inhibitor of transcriptional response to 1,25(OH)_2_D [89]. Thus, sumoylation results in the decreased transcriptional activity of VDR. Moreover, it was found that sentrin/SUMO specific protease 1 and 2 (SENP1 and SENP2) can reverse modifications of VDR by SUMO2. This led to potentiated ligand-mediated transactivation of VDR. These fine-tuning effects are cell line-dependent. While the most achieved potent modulatory effects were described in Caco-2 and HEK-293 cells, the 1,25(OH)_2_D signal in MCF-7 cells was unaffected by tested SENP [90]. The same research group identified lysine 91 (localized in potential site II (non-consensus) acceptor site; VDR lacks a true SUMO consensus sequence) as a minor SUMO acceptor site within VDR [90].

### Acetylation

Reaction that introduces an acetyl functional group into a molecule is termed acetylation. It is common modification in most nuclear receptors, including PXR [91], AR or ERα [92]. Surprisingly, there is no clear proof of VDR protein acetylation to this date. Indirect evidence was brought by Stone *et al*., who demonstrated potentiation of VDR activity by resveratrol, NAD-dependent deacetylase sirtuin-1 (SIRT1) activator [93]. Resveratrol and 1,25(OH)_2_D have synergistic effect on VDR-RXR heterodimerization and VDR-mediated transcription. Comparison of wild-type VDR and ligand-binding domain mutant VDR has shown that response to 1,25(OH)_2_D was severely depressed, while the response to resveratrol was only moderately attenuated. This implies an indirect effect of resveratrol to VDR [93]. Moreover, phylogenetic analysis of PXR and VDR suggests that lysine residue in VDR protein equivalent to lysine 109 in PXR might undergo acetylation similarly as it was demonstrated in PXR [91].

## DISCUSSION

This review provides basic information about post-transcriptional and post-translational modifications of the VDR. It is surprising, that transcription factor, which regulates more than 1000 genes [94], has not been well studied in this research area.

In the case of post-transcriptional regulation, only two miRNAs regulating the amount of VDR were found. Interestingly, both miRNAs can reduce VDR on the protein level with no effect on the mRNA level, indicating blockage of translation rather than increased mRNA degradation. However, more experiments are required to elucidate the regulation of VDR *via* miRNAs and to show correlating effects of miRNAs decrease with increased sensitivity to 1,25(OH)_2_D anticancer activity. Thus, monitoring the level of miRNAs mentioned above could be used as a biomarker in order to justify vitamin D supplementation for supportive treatment in anticancer therapies. Additionally, searching for the therapeutics decreasing the expression of these miRNAs might be useful for sensitization of cancers for consequent vitamin D treatments. This was reported for celecoxib, a nonsteroidal anti-inflammatory drug (NSAID) capable of restoring the level of miR-29c, which is downregulated in gastric cancer tissues. Increased expression of miR-29c induced apoptosis in gastric cancer cells *via* suppression of oncogene Mcl-1 [95].

To this date, only three major post-translational modifications of VDR (phosphorylation, sumoylation and ubiquitination) have been demonstrated. The last two modifications were identified recently and it will take more time for precise understanding how these modifications affect the VDR activity or biology. Other PTMs, such as acetylation, glycosylation, palmitoylation or methylation have not been proven in VDR protein yet. However, some of these modifications were demonstrated in other nuclear receptors, including AR, liver x receptor (LXR) or ER-α [92, 96, 97].

Since VDR holds many posts in human biology, deciphering the role of PTMs in context of proper VDR signaling or improved pharmacotherapy is crucial. Some of the modifications like sumoylation, ubiquitination or acetylation take place on nitrogen atom of lysine residue. Thus, these PTMs create interface for protein binding as a “gain of function” mechanism and may modulate the protein function, such as of protein-protein interaction, DNA binding, transcriptional activity, stability and subcellular localization. In addition, they greatly alter the electrostatic properties of a protein by neutralizing the positive charge of the lysine residue. Furthermore, they disrupt a formation of hydrogen bonds on lysine side-chains. Moreover, type of binding for instance to either lysine 48 (K48) or lysine 63 (K63) in ubiquitin determines either a recognition signal for 26S proteasome degradation (K48) or a molecular platform for protein-protein interaction or receptor trafficking (K63) [98].

Possible benefits of knowledge of this type of PTM could be found among patients with plasmablastic lymphoma or myeloma cells. It was demonstrated that vitamin D inhibited the growth of these cells and that *FokI* variant of VDR displayed greater growth inhibition [99]. Since inhibition of proteasome increases the VDR protein together with higher stability of VDR in the presence of ligand, especially multiple myeloma (MM) patients, treated with proteasome inhibitor bortezomib, might greatly benefit from vitamin D supplementation. Moreover, this supplementation could mitigate peripheral neuropathy observed in 25(OH)D-deficient MM patients [100].

Genetic polymorphisms affect most of the genes, making VDR gene no exception. These changes in the core DNA sequence may result in the different amino acid sequence in final protein. Consequently, this may affect resulting behavior of VDR as it was observed in the case of mutated serine 51 [54, 55]. In addition, different levels of VDR expression as well as PTMs-creating enzymes in different tissues may highlight the importance of these PTMs in physiology/pathology of particular tissue. This seems to be true especially for cancer. It was demonstrated that carcinogens- or DNA double strand breaks-triggered ATM kinase positively stimulates activity of VDR, which in turn induces ATM gene expression [66]. This creates a positive feedback loop and contributes to chemopreventive mechanism of vitamin D. Thus, genotyping the patients with cancer for mutations at serine residues 208/222 of VDR might be used for exclusion of these patients from vitamin D-directed anti-cancer therapy.

Another example represents the Alzheimer's disease (AD), in therapy of which some studies suggested protective role of vitamin D [101, 102]. Moreover, it was demonstrated that vitamin D prevented amyloid-beta cytotoxicity, one of the major components of AD pathology, in primary cortical neurons [70]. This opens a new strategy in treatment or at least in slowing down the progression of this disease. Moreover, the sensitization of this process could be achieved by development of specific PKA inhibitors, especially those targeting catalytic (Cβ) or regulatory subunit (RIIβ) of PKA, which are expressed at high levels in nervous system [103].

In general, the field of PTMs of VDR represents great knowledge that can be used for understanding of physiological impact of this receptor. Moreover, this knowledge can be used at least for preventive purposes of some pathological states. However, every step we make leads us to a new unexplored territory that is usually not much clear in terms of consequences it may have. Many studies must be conducted in order to better understand all the connections and consequences between post-transcriptional/−translational modifications of VDR and its activity.

## Abbreviations

Vitamin D_3_: cholecalciferol
Vitamin D_2_: ergocalciferol
VDR: vitamin D receptor
1,25(OH)_2_D-1α,25: dihydroxyvitamin D_3_ (calcitriol)
1α,25: dihydroxyvitamin D_2_
VDRE: vitamin D responsive element
RXR: retinoid X receptor
miRNA: microRNA
MRE 125b: miR-125b recognition element
3´-UTR: 3´-untranslated region
PKC: β-protein kinase C-β
CK-II: casein kinase II
SUMO: Small Ubiquitin-like modifier
SENP: sentrin/SUMO specific protease
PIAS4: protein inhibitor of activated STAT
CDK11^p58^: cyclin-dependent kinase 11, 58 kDa protein
PKA: protein kinase A
AF-2: activation function 2
8 bromo-cAMP: 8-bromoadenosine cyclic 3´,
5´: monophosphate
SUG1: suppressor for gal 1
NR: nuclear receptor.
